# Evaluation of peach tolerance after one year of sublingual immunotherapy with LTP (Pru p 3) in allergic patients sensitises to food by LTPs

**DOI:** 10.1186/2045-7022-5-S3-O14

**Published:** 2015-03-30

**Authors:** Gador Bagos, Francisca Gómez, María Salas, Paloma Campo, Luisa Galindo, María Jose Torres, Cristobalina Mayorga, Lucía Jimeno, Miguel Blanca

**Affiliations:** 1 Allergy Service, Carlos Haya Hospital, Malaga, Spain; 2 Research Laboratory, Carlos Haya Hospital-FIMABIS, Malaga, Spain; 3 ALK-Abello, Madrid, Spain, Madrid, Spain

## Rationale

In Southern Europe Pru p3 is the primary sensitizer of plants fruit and responsible for severe reactions. Specific immunotherapy (SIT) brings a new perspective to treat those patients. The aim was to evaluate the effect of sublingual immunotherapy (SLIT) to peach in allergic patients with systemic symptoms.

## Methods

Forty-six peach allergic patients confirmed as positive by skin test, ImmunoCAP IgE and/or a double-blind placebo-controlled food-challenge (DBPCFC) were included. After one year of treatment we evaluated peach tolerance with DBPCFC using 5, 40, and 120 g of fresh peach with a mixture of yogurt, orange juice, coffee dried, coconut, and oatmeal flakes. Placebo meals consisted of the same ingredients, without fresh apple.

## Results

From the total group, 21 patients (45%) had anaphylaxis and 25 (55%) urticaria and/or angioedema. The 82,6% showed sensitization to other plant foods proteins and 69,5% showed sensitization to pollens. The DBPCFC was performed in 20 patients who completed the first year of SLIT. The 90% patients had good tolerance to peach during DBPCFC as well as fresh peach at home. Only 2 patients (10%) presented reactions. One patient suffered an anaphylactic reaction after 45 g of the active mixture, and other patient suffered angioedema after ingest complete peach at home.

## Conclusion

These results showed a high percentage of patients who tolerated peach after one year receiving SLIT. There were no differences in the clinical patterns between the patients with good response and those patients with reactions.

